# Prognostic impact of miR-34b/c DNA methylation, gene expression, and promoter polymorphism in HPV-negative oral squamous cell carcinomas

**DOI:** 10.1038/s41598-022-05399-1

**Published:** 2022-01-25

**Authors:** Gordana Supic, Debora Stefik, Nemanja Ivkovic, Ahmad Sami, Katarina Zeljic, Sasa Jovic, Ruzica Kozomara, Danilo Vojvodic, Srboljub Stosic

**Affiliations:** 1 Faculty of Medicine, Military Medical Academy, University of Defense, Belgrade, Serbia; 2 Institute for Medical Research, Military Medical Academy, Crnotravska 17, 11002 Belgrade, Serbia; 3 Cellular and Molecular Radiation Oncology Laboratory, Department of Radiation Oncology, Universitaetsmedizin Mannheim, Medical Faculty Mannheim, Heidelberg University, Mannheim, Germany; 4 Faculty of Biology, University of Belgrade, Belgrade, Serbia; 5 Clinic for Maxillofacial Surgery, Military Medical Academy, Belgrade, Serbia

**Keywords:** Cancer, Genetics, Molecular biology, Oncology

## Abstract

Micro RNAs (miRNAs) have a key role in gene expression regulation in cancer. The aim of the current study is to evaluate the prognostic value of miR-34b/c promoter hypermethylation, gene expression, and polymorphism in HPV-negative oral squamous cell carcinomas (OSCC). MiR-34b/c promoter hypermethylation and pre-miR-34b/c polymorphism rs4938723 were evaluated in tumor tissues of 148 patients, and miR-34b expression in 123 HPV-negative OSCC. For risk assessment, the control group was comprised of 175 healthy individuals. MiR-34b/c promoter hypermethylation was determined by methylation-specific PCR. Gene expression, genotyping and HPV screening was assessed by Q-PCR. The data from our hospital cohort indicated that miR-34b/c DNA methylation was associated with nodal status (p = 0.048), and predicted the shorter overall survival of HPV-negative OSCC patients (p = 0.008). Down-regulated miR-34b/c expression was associated with smoking (p = 0.047), alcohol use (p = 0.009), stage (p = 0.025), recurrences (p = 0.000), and a poor survival (p = 0.00029). Median values of miR-34b expression were significantly lower in advanced stages III/IV as opposed to stage I/II, p = 0.006, and in nodal positive vs negative patients (p = 0.045). TCGA data also indicated that tumors with stage I–III expressed significantly higher levels of miR-34b, compared to tumors with stage IV (p = 0.035), Low miR-34b/c expression was associated with poor survival in smokers (p = 0.001) and patients with tongue carcinomas (p = 0.00003), and TCGA analysis confirmed these findings although miR-34b expression and miR-34b/c methylation were not associated with survival outcome in the whole TCGA cohort. A significant negative miR-34b/c expression–methylation correlation was observed in our hospital cohort (p = 0.017) and in TCGA cohort. Pre-miR-34b/c polymorphism was not associated with oral cancer risk. Our findings indicate that miR-34b/c hypermethylation and low miR-34b expression could promote the progression and predict the poor prognosis for HPV-negative OSCC, which suggests miR-34b/c as a promising biomarker and therapeutic target for OSCC in the future.

## Introduction

Despite various advances in the last decades in molecular characterization, early detection, and treatment modalities, Oral Squamous Cell Carcinoma (OSCC) remains one of the most aggressive subset of the Head and Neck Squamous Cell Carcinoma (HNSCC)^1^. Poor prognosis and the 5-year survival of OSCC at approximately 50% is linked to the high incidence of lymph node metastasis, and loco-regional recurrences^1^. A rising incidence of OSCC regardless of the reduced smoking and alcohol consumption in recent years and a relatively high proportion of OSCCs (15–30% of HNSCC) in the developed world^2^ requires further molecular characterization and the identification of novel prognostic biomarkers. While smoking and alcohol drinking are established risk factors, human papillomaviruses (HPV) infection has been increasingly recognized as one of the key risk factors for OSCC. HPV status has been included for stratification of oral cancer patients in terms of outcome and response to therapy, where patients with HPV-negative OSCC display significantly worse survival and poorer response to therapy than those with HPV-positive cancers^2^. Recent studies revealed the associations between HPV status and promoter methylation in oral carcinomas^3^, which indicates that epigenetic modifications might be responsible for the distinctive clinical behavior of HPV-negative OSCC.

Aberrant epigenetic modifications, changes in gene expression that occur without alterations in the DNA sequence, has emerged as one of the hallmarks of oral carcinogenesis. The major mechanisms include DNA methylation, histone modifications, and non-coding RNAs. DNA methylation involves the covalent addition of a methyl group to the cytosine of CpG dinucleotides within CpG islands. In normal cells, CpG islands are unmethylated as opposed to cancer cells, where tumor suppressor genes are commonly silenced by hypermethylation and associated with transcriptional repression^4^.

Amongst various classes of non-coding RNAs, microRNAs (miRNAs), with an average of 22 nucleotides in length, are one of the major posttranscriptional regulators of gene expression^5^. MicroRNAs regulate the expression of over 60% of human genes by binding to 3′-UTR, to selectively regulate the cleavage of target mRNAs and/or inhibit or activate translation^6^. Based on its target genes, microRNAs may act as oncogenes or tumor suppressors consequently playing a critical role in cancer development and progression^5^. However, several miRNAs, including the miR-34 family, appear to have a controversial dual role either behaving as oncogenes or tumor suppressors, depending on the tumor type and presence of specific targets^7,8^.

The miR-34 family members exert key roles in a number of cancer-associated processes, including apoptosis and cell proliferation^9,10^, epithelial to mesenchymal transition^11,12^, angiogenesis^12^, and migration, invasion, and metastasis^12–14^. The genes coding for miR-34b and miR-34c are clustered as homologous genes at chromosome 11q23 and have a common primary transcript, pri-miR-34b/c^15^. miR-34b/c gene is located in a CpG island region and silenced by DNA methylation in a variety of cancer types^8^, including oral cancer^16,17^, esophageal cancer^18,19^, colorectal^20^, breast^21^, and non-small cell lung cancer^22^. There are conflicting findings of miR-34b/c expression in various carcinomas. While miR-34b acts as a tumor suppressor and is down-regulated in colorectal, gastric, and breast carcinoma^20,21,23^, in HNSCC miR-34b is commonly over-expressed and exerts oncogenic potential^24^. Moreover, the miR34b gene shows expression divergence in HNSCC of different anatomical sites, in oral carcinomas as opposed to carcinomas of the larynx and hypopharynx^25–27^. Recent findings indicate that the expression of miR-34b is decreased in metastatic OSCC compared with nonmetastatic ones^28^, indicating that this microRNA could play an important role in the metastasis of OSCC. Moreover, genetic polymorphism rs4938723, located in the promoter of the pre-miR-34b/c gene, was previously associated with an increased risk of hepatocellular carcinoma^14^, but also with the decreased susceptibility to esophageal cancer, leukemia, and colorectal cancer^29,30^.

Thus, the aim of the current study is to investigate the prognostic value of miR-34b/c promoter DNA methylation, polymorphism rs4938723 in the pri-miR-34b/c, and miR-34b gene expression in patients with HPV-negative oral carcinomas. Our integrative approach in evaluating the prognostic value of miR-34b/c could potentially yield important insights into its biological and clinical relevance in oral carcinomas.

## Results

### MMA hospital cohort miR-34b/c promoter hypermethylation analysis

**Figure 1:**
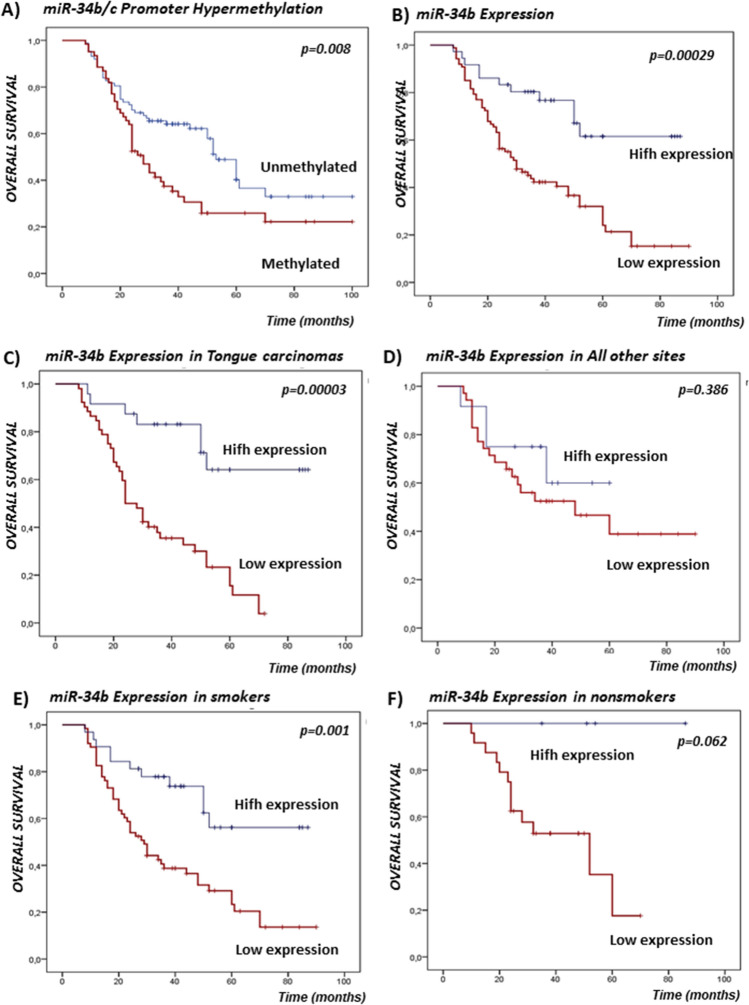
Kaplan–Meier curves with log-rank test for overall survival (OS) of the OSCC patients cohort. (**A**) Survival according to the miR-34b/c promoter hypermethylation status. (**B**) Survival according to the miR-34b gene expression. (**C**) Survival according to the miR-34b expression in tongue carcinomas patients. (**D**) Survival according to the miR-34b expression in patients with OSCC of all other sites. (**E**) Survival according to the miR-34b expression in smokers. (**F**) Survival according to the miR-34b expression in non-smokers.

**Figure 2:**
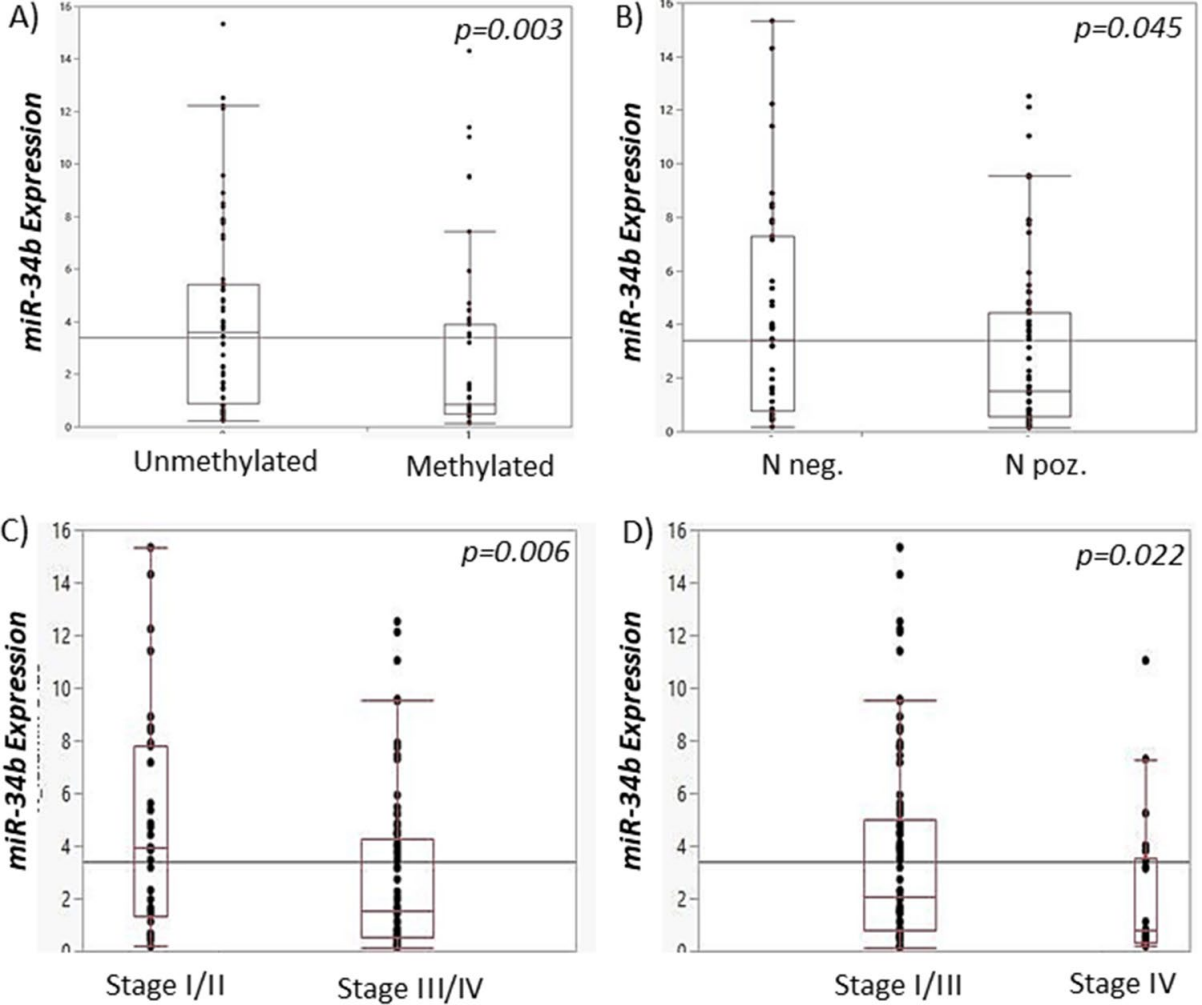
miR-34b/c methylation and miR-34b expression in the MMA hospital cohort (**A**) Association of miR-34b/c methylation and miR-34b expression (**B**) miR-34b expression and nodal status (**C**) miR-34b expression in stage III/IV compared to stage I/II tumors (**D**) miR-34b expression in stage IV compared to stage I–III tumors.

DNA methylation of miR-34b/c gene promoter was detected in 61/148 (41.22%) of samples. miR-34b/c promoter methylation was associated with nodal status (p = 0.048), Table 1, and significantly predicted the shorter overall survival of HPV-negative OSCC patients (p = 0.008, log-rank test) (Fig. 1A). According to Cox hazard regression analysis, patients with the hypermethylated miR-34b/c promoter had a 2170 times higher risk of poor overall survival (p = 0.004), compared to patients with unmethylated miR-34b/c, but not independently of recurrences and nodal status (Table 2). We observed significant association of miR-34b/c promoter hypermethylation status and miR-34b expression (p = 0.0035) (Fig. 2A).

**Table 1:** Association of miR-34b/c promoter methylation, gene expression and rs4938723 polymorphism with demographic and clinico-pathohistological features of OSCC patients.

Variables		miR-34b/c methylation, N = 148pts		miR-34b expression, N = 123pts		miR-34b/c polymorphism, N = 148pts				
		U	M	Low	High	TT		TC		CC
Sex	Male	62	48	68	28	45		46		19
	Female	25	13	19	8	16		14		8
	*p*	0.309		0.963		0.817				
Age (≥ median)	< 58	49	38	48	25	39		32		16
	≥ 58	38	23	39	11	22		28		11
	*p*	0.467		0.143		0.495				
Smoking	Never/Ex	17	18	24	4	12		18		5
	Current	70	43	63	32	49		42		22
	*p*	0.160		**0.047**		0.322				
Alcohol	Low	45	29	33	23	27		36		11
	High	42	32	54	13	34		24		16
	*p*	0.616		**0.009**		0.127				
Cancer site	Tongue	51	39	52	24	39		31		20
	Mouth floor	9	8	10	5	5		12		0
	Palate	5	3	2	1	2		4		2
	Buccal mucosa	3	3	6	0	3		0		3
	Gingiva	4	0	2	2	2		2		0
	Overlapping lesion of mouth	15	8	15	4	10		11		2
	*p*	0.573		0.511		0.050				
Histological grade	1/2	75	51	70	34	50		52		24
	3	12	10	17	2	11		8		3
	*p*	0.662		0.051		0.639				
Nuclear grade	1/2	71	46	66	32	48		45		24
	3	16	15	21	4	13		15		3
	*p*	0.362		0.102		0.337				
Stage	I/II	29	12	19	15	19		14		8
	III/IV	58	49	68	21	42		46		19
	*p*	0.068		**0.025**		0.612				
Tumor size	T1/2	54	40	51	27	40		40		14
	T3/4	33	21	36	9	21		20		13
	*p*	0.663		0.086		0.377				
Nodal status	N−	35	15	27	16	26		12		12
	N+	52	46	60	20	35		48		15
	*p*	**0.048**		0.156		**0.014**				
Recurrences	No	34	23	25	24	23		22		12
	Yes	53	38	62	12	38		38		15
	*p*	0.866		**0.000**		0.777				

*U* Unmethylated, *M* methylated.

miRNAs High/Low expression defined according to cutoff (3.58) derived from ROC analysis.

Significant values, p < 0.05, are bolded.

**Table 2:** Hazard risk analysis of prognostic factors in relation to overall survival (OS) according to Cox regression analysis, in OSCC patients.

Cox regression	Variables	*OS*	
		HR [95% CI]	*p*
Univariate analysis	Sex	1.548 [0.920–2.604]	0.100
	Age (≥ median)	1.403 [0.911–2.162]	0.124
	Smoking	1.431 [0.831–2.464]	0.196
	Alcohol	1.471 [0.957–2.259]	0.078
	Cancer site	1.025 [0.910–1.154]	0.685
	Histological grade	1.328 [0.963–1.832]	0.084
	Nuclear grade	1.479 [1.088–2.011]	**0.012**
	Stage	2.847 [2.015–4.023]	**0.000**
	Tumor size	1.528 [1.241–1.882]	**0.000**
	Nodal status	2.675 [1.572–4.552]	**0.000**
	miR-34b/c methylation	1.746 [1.140–2.673]	**0.010**
	miR-34b/c expression	3.056 [1.603–5.825]	**0.001**
	Pre-miR-34b/c rs4938723	0.864 [0.641–1.165]	0.337
	Recurrences	7.796 [4.185–14.523]	**0.000**
Multivariate analysis	Recurrences	10.643 [5.199–21.790]	**0.000**
	N	2.229 [1.228–4.046]	**0.008**
	miR-34b/c methylation	2.170 [1.282–3.671]	**0.004**

*OS* Overall survival, *HR* hazard ratio, *CI* confidence interval.

miRNA expression dichotomized as Low and High according to ROC analysis of normalized miR expression.

Significant values, p < 0.05, are bolded.

### MMA hospital cohort miR-34b expression analysis

ROC curve, assessed to evaluate the predictive value and the optimal cutoff point for miR-34b over-expression, demonstrated that miRNA-34b expression might be an indicator of poor outcome in the OSCC (ROC-AUC 0.654, [0.552–0.756], p = 0.004), and that fold change expression of 4.54 is an optimal cutoff point with highest prognostic performance (Supplementary Fig. 1).

Forty patients (36/123. 29.27%) had down-regulated miR-34b expression. Low miR-34b expression accociated with smoking (p = 0.047), alcohol use (p = 0.009), advanced stage III and IV (p = 0.025), and recurrencies (p = 0.000) (Table 1). HPV-negative OSCC patients with the decreased miR-34b tumor expression are associated with reduced overall survival (p = 0.00029, log-rank test) (Fig. 1B). Furthermore, low miR-34b/c expression was significantly associated with poor survival in patients with tongue carcinoma (p = 0.00003) (Fig. 1C), compared with all other sites (p = 0.386) (Fig. 1D). Also, low miR-34b/c expression was significantly associated with poor survival in smokers (p = 0.001) (Fig. 1E), compared with non-smokers (p = 0.0.062) (Fig. 1F). Median values of miR-34b expression were lower in lymph node-negative patients than in node-positive patients (p = 0.045) (Fig. 2B). In addition, median values of miR-34b expressions were significantly lower in advanced stages III and IV as opposed to stage I and II, p = 0.006 (Fig. 2C), and in stage IV compared to stage I to III, p = 0.022 (Fig. 2D).

### MMA hospital cohort pre-miR-34b/c polymorphism genotyping

Pre-miR-34b/c promoter polymorphism rs4938723 did not significantly associate with overall survival (p = 0.165), but associated with nodal status (p = 0.014) (Table 1). Logistic Odds Ratio analysis, adjusted for age and gender, showed that pre-miR-34b/c polymorphism rs4938723 is not significantly associated with the susceptibility to oral cancer for TC genotype (OR 0.915, p = 0.708), and CC genotype (OR 1.780, p = 0.165), compared to referent TT genotype, or in a dominant model (OR 1.865, p = 0.116), (Supplementary Table 1).

To identify the relationship between miR-34b gene expression, miR-34b/c promoter hypermethylation, and pre-miR-34b/c polymorphism, we performed a correlation analysis. The miR-34b expression was negatively correlated with its promoter hypermethylation (Spearman’s correlation coefficient = − 0.215, p = 0.017). We did not observe any significant polymorphism-expression correlation (Spearman’s coefficient = − 0.071, p = 0.438), or polymorphism-methylation correlation (Spearman’s coefficient = − 0.029, p = 0.725).

### TCGA cohort miR-34b/c promoter hypermethylation analysis

**Figure 3:**
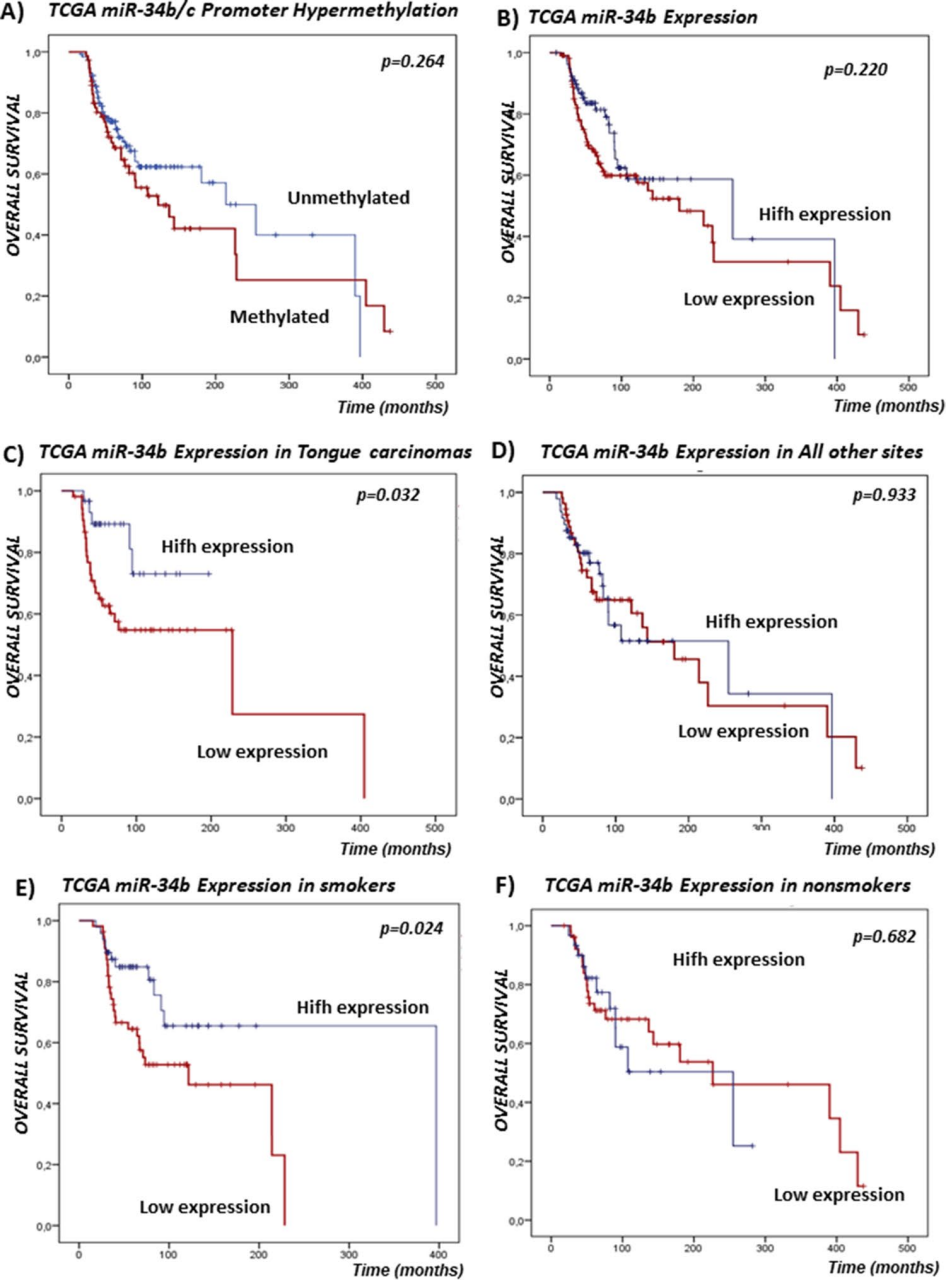
Kaplan–Meier curves with log-rank test for overall survival (OS) of the TCGA cohort. (**A**) Survival according to the miR-34b/c promoter hypermethylation status. (**B**) Survival according to the miR-34b gene expression. (**C**) Survival according to the miR-34b expression in tongue carcinomas patients. (**D**) Survival according to the miR-34b expression in patients with OSCC of all other sites. (**E**) Survival according to the miR-34b expression in smokers. (**F**) Survival according to the miR-34b expression in non-smokers.

**Figure 4:**
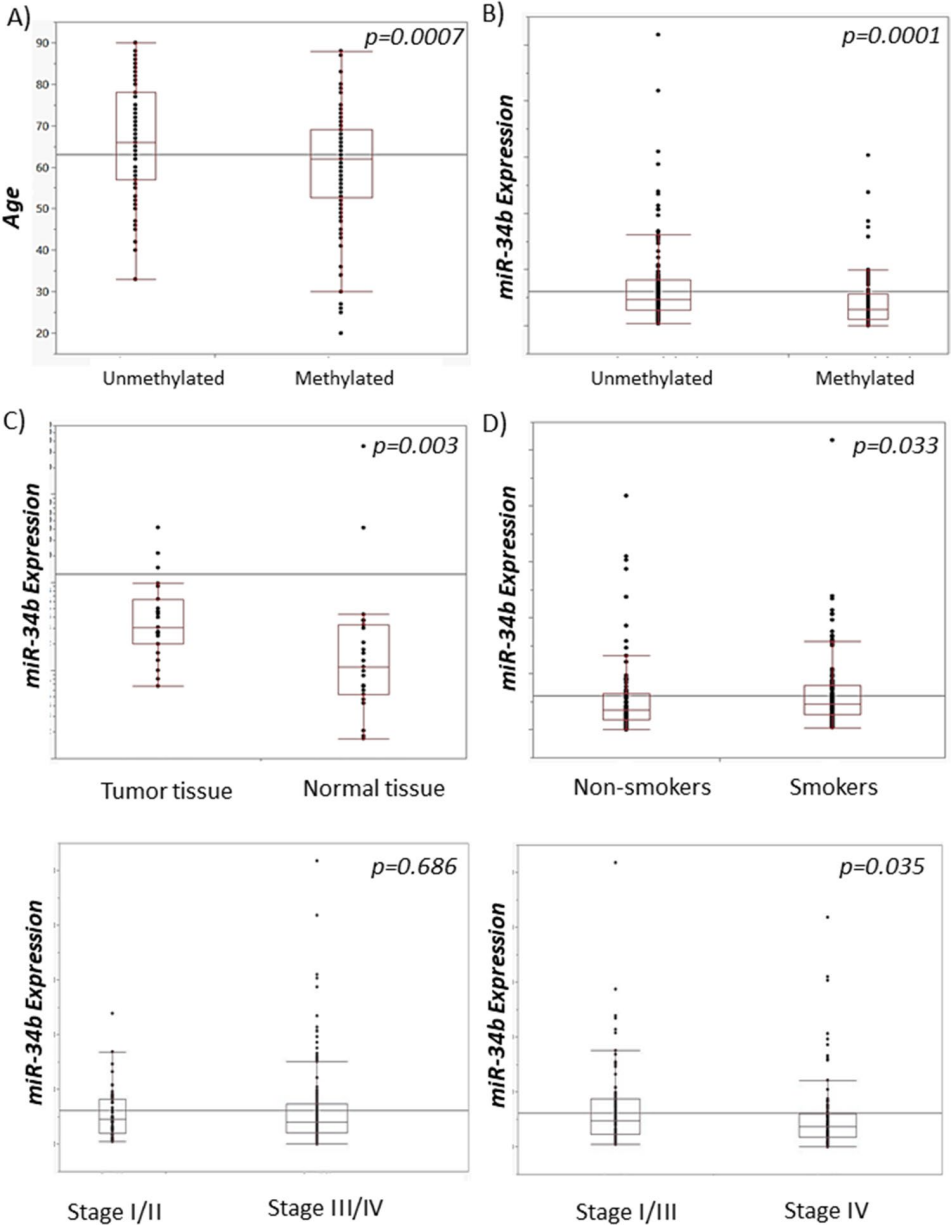
miR-34b/c methylation and miR-34b expression in the TCGA cohort (**A**) Association of methylation status and age (**B**) Association of miR-34b/c methylation and miR-34b expression (**C**) miR-34b expression levels (log scale): tumor vs normal tissue (**D**) miR-34b expression and smoking status (**E**) miR-34b expression in stage III/IV compared to stage I/II tumors (**F**) miR-34b expression in stage IV compared to stage I–III tumors.

The K means Clustering analysis has clearly separated two groups of samples (Supplementary Fig. 2). According to the analysis of available TCGA data, 138 tumor samples were hypomethylated, while 87 tumor samples was hypermethylated. miR-34b/c promoter hypermethylation status did not associate with survival in TCGA cohort (p = 0.264) (Fig. 3A). For 44 patients with available data across matched tumor and normal tissue samples, tumor tissue samples were associated with hypermethylation status of promotor region compared to normal tissue samples of the same patients, (p = 0.0212). From normal tissue samples, only one sample was hypermethylated and 21 was hypomethylated. In addition, patients with hypermethylated region of interest in tumor tissue were significantly older compared to patients with hypomethylated status of tumor tissue (N = 225 patients, p = 0.0007, Mann–Whitney U test) (Fig. 4A). We observed significant association of miR-34b/c promoter hypermethylation status and lower miR-34b expression in TCGA cohort (p = 0.0001) (Fig. 4B). Expression levels of miR-34b showed negative correlation with the beta values of 12 analyzed CpG sites (p < 0.001) (Supplementary Fig. 3).

### TCGA cohort miR-34b expression analysis

miR-34b expression did not associate with survival in TCGA cohort (p = 0.220) (Fig. 3B). However, stratification by tumor site revealed that low miR-34b expression was associated with poor survival in patients with the tongue carcinomas (p = 0.032) (Fig. 3C), compared with all other sites (p = 0.933) (Fig. 3D). Also, stratified by smoking status our analysis indicated that low miR-34b expression was associated with poor survival of smokers (p = 0.024) (Fig. 3E), compared to non-smokers (p = 0.682) (Fig. 3F).

The statistically significant difference in mir-34b expression levels has been detected between normal and tumor tissue, in mached samples from the same patients (N = 46, p = 0.0034) (Fig. 4C). Tumor samples from patients who consume cigarettes or former smokers had significantly higher levels of mirR-34b compared to non-smokers (N = 223 p = 0.033) (Table 3). Advanced stages III and IV compared to stage I and II did not show differences in miR-34b expression, p = 0.686 (Fig. 4C). However, tumors with stage I–III expressed significantly higher levels of miR-34b, compared to tumors with stage IV (N = 204, miR34b: p = 0.035) (Fig. 4D).

**Table 3:** The association of miR-34b/c promoter methylation, and miR-34b gene expression with demographic and clinico-pathohistological features of validation TCGA cohort.

Variables		miR-34b expression median (25–75%)	miR-34b/c methylation	
			U	M
Sex N = 225	Male	3.904 (1.963–7.469)	93	51
	Female	4.210 (2.620–6.880)	45	36
	*p*	0.280	0.201	
Age (> median) N = 225	< 64	4.17 (2.329–7.143)	74	43
	≥ 64	3.970 (2.092–7.733)	50	58
	*p*	0.793	**0.0113**	
Smoking N = 225	Never/Ex	3.500 (1.1782–6.403)	56	46
	Current	4.59 (2.672–7.930)	82	41
	*p*	**0.033**	0.076	
Alcohol N = 220	Low	4.120 (2.391–7.680)	44	32
	High	4.080 (1.973–7.165)	91	53
	*p*	0.700	0.200	
Cancer site N = 225	Tongue	3.802 (2.444–6.497)	67	30
	Mouth floor	5.634 (2.265–9.503)	29	20
	Palate	1.827 (0.139–5.587)	1	3
	Buccal mucosa	7.129 (2.343–16.29)	11	4
	Gingiva	2.203 (1.266–5.550)	3	3
	Overlapping lesion of mouth + lip	4.038 (1.780–6.203)	27	27
	*p*	0.0561	0.432	
Stage N = 205	I/II	4.610 (1.953–8.217)	31	23
	III/IV	4.0663 (2.091–7.329)	95	56
	*p*	0.686	0.427	
	I–III	4.740 (2.298–8.762)	57	38
	IV	3.684 (1.813–6.028)	69	41
	*p*	**0.035**	0.516	
Tumor size N = 211	T1/2	4.173 (2.114–7.733)	62	31
	T3/4	4.024 (2.033–15.851)	68	50
	*p*	0.675	0.201	
Nodal status N = 192	N−	4.610 (2.068–8.216)	46	41
	N+	3.801 (2.049–6.972)	73	32
	*p*	0.220	**0.025**	

Significant values are in bold.

*U* Unmethylated, *M* Methylated.

Mann–Whitney *U* test was used for comparison expression data with clinico-pathohistological features.

## Discussion

Oral Squamous Cell Carcinoma is the one of the most aggressive types of cancer, characterized by the poor prognosis and the high incidence of metastasis, and loco-regional recurrences^1^. Furthermore, patients with HPV-negative OSCCs have a worse overall survival compared to the HPV‐positive ones^31,32^. The HNSCC and OSCC, derived from different sites of the upper aerodigestive tract, show significant heterogeneity in clinical behavior and microRNAs expression patterns^26^, and this expression divergence was also observed for miR34-b/c^25–27^. While miR-34b/c has been suggested as a tumor suppressor in a number of tumor types^11,21,23,33–35^, including oral cancer^16,17^, it has been reported to be over-expressed in head and neck carcinomas^18^.

In light of a significantly worse 5-year overall survival of HPV-negative compared to HPV-positive OSCC patients, we reasoned to examine the miR-34b/c in a subset of HPV-negative oral carcinomas. Our findings indicate that miR-34b/c could promote the progression and poor prognosis of HPV-negative OSCC patients. The miR-34b/c promoter methylation was associated with nodal status, and shorter overall survival of oral cancer patients, and we observed a significant negative miR-34b expression–methylation correlation. These results have been confirmed in the TCGA validation cohort. The data from our hospital cohort indicate that patients with the decreased miR-34b tumor expression had significantly reduced overall survival. Furthermore, stratified analysis according to smoking and tumor localization show that low miR-34b/c expression was significantly associated with poor survival in smokers and patients with tongue carcinomas, which was also confirmed in the validation TCGA cohort. Pre-miR-34b/c polymorphism rs4938723 was not significantly associated with oral cancer risk in our study.

Our study demonstrating association of miR-34b/c low expression and promoter hypermethylation with shorter overall survival of OSCC patients is in line with previous findings in various solid carcinomas^17,19–22,36^. miR-34b/c gene is positioned within CpG island and is silenced through DNA methylation of its promoter, which identified miR-34b/c as tumor-suppressive miRNAs^37^. Our findings are in agreement with a previous study reporting that miR-34b promoter hypermethylation is relatively common and correlated with decreased mir-34b expression and poor overall survival in OSCC^16^. Also, the expression of miR-34b in OSCC cell lines inversely correlate with DNA methylation status and can be restored by the demethylating agent 5-aza-2′-deoxycytidine^16^, demonstrating that miR-34b is epigenetically silenced during oral carcinogenesis.

In line with our findings of significant association of miR-34b/c promoter hypermethylation and miR-34b expression with lymph node metastases and stage, a number of studies indicate that DNA methylation and decreased expression of miR-34b due to its promoter DNA methylation contributes to the cancer metastases^13,14,37^. Recent findings demonstrate that the expression of miR-34b is decreased in metastatic oral carcinomas as opposed to nonmetastatic ones and that miR-34b could play an important role in the OSCC metastasis by regulating matrix metalloproteinases^28^.

Previous studies reported that miR-34b is prominently downregulated and exert a tumor suppressor role in a number of tumor types^11,12,21,23,33–35^, including esophageal^27^, nasopharyngeal^38^, and oral carcinomas^16,17^. However, other studies on HNSCC^18,25,27^ and a study on tongue carcinoma^39^, a subset of OSCC, reported that miR-34b/c is over-expressed and could have oncogenic properties, which point to the potential dual role of miR-34b/c, oncogenic and tumor-suppressive, depending on the tumor subtype and molecular context. HNSCC is a heterogeneous group of distinct malignancies derived from diverse anatomical sites and related to different risk factors, with diverse molecular features and clinical outcomes that may have different miRNA signatures. Oral cavity tumors show different patterns and more variations in gene expressions than larynx and hypopharynx carcinoma, the other two major subsets of HNSCC^26^. These findings support the hypothesis that molecularly defined subtype classification in HNSCC could improve patient selection and the development of appropriate therapeutic strategies.

Although pre-miR-34b/c polymorphism was previously associated with increased risk in hepatocellular carcinoma^29^ and, conversely, with a decreased predisposition to leukemia, colorectal, HNSCC^29,30^, and oral cancer^40^, our study did not confirm this. Studies with a larger sample size with the inclusion of gene–gene or gene–environment interactions should be carried out to further elucidate the role of miR-34b/c rs4938723 polymorphism in oral cancer risk.

To our knowledge, miR-34b/c methylation and miR-34b expression has not been previously investigated in the HPV-negative subset of oral carcinomas. Only one study investigated miR-34a and miR-34c expression in HPV-negative and HPV-positive oropharyngeal carcinomas, and identified miR-34c as significantly deregulated in HPV-negative OSCC^41^. HPV-negative OSCC is characterized by the accumulation of genetic changes in a number of tumor suppressor genes, including TP53, RB1, CDKN2a, NF‐kappaB, and STAT3^42^, induced by smoking and alcohol consumption rather than viral infection. Mutated TP53 and other tumor suppressor genes cannot be re-activated after chemoradiation to initiate the apoptosis of cancer cells, which might be associated with a worse prognosis and decreased sensitivity to chemoradiation therapy of HPV-negative OSCC in contrast to the HPV‐positive ones^31,32^. Several studies revealed the differences in promoter methylation profiles between HPV-positive and HPV-negative oral carcinomas^3^, indicating that epigenetic modifications may be responsible for the distinctive clinical behavior of HPV-negative OSCC. However, a study on esophageal cancer did not find an association between miR-34b/c methylation and HPV infection status^19^, suggesting that HPV infection and miR-34b/c methylation may act independently to influence the cancer risk.

It has been reported that the miR-34 family target a number of genes involved in cell cycle progression, including TP53, MDM4, CDK6, CDK4, and MYC^9,10,43^. Expression of the miR-34 family is inversely associated with the TP53 mutation status in HNSCC^25^. The miR-34a and miR-34b/c appear to be the most prevailing p53-induced miRNAs^43^. In response to DNA damage, hypoxia, and oncogenic stimuli, p53 activates the miR-34 family and induces cell cycle G1-arrest and/or apoptosis, and inhibits cell proliferation^9,10^. On the other hand, the p53-mediated up-regulation of miR-34b provides a feedback loop in the p53 master regulatory network in cancer, by down-regulating Met, which subsequently controls its downstream signaling molecules, p53 and Mdm2^44^. Furthermore, miR-34b is involved in an EMT by targeting SNAIL^11,45,46^, mesenchymal markers (Cdh2 and Fn1) and epithelial markers (Cldn3, Dsp, and miR-200)^11^. In thyroid carcinoma, miR-34b/c act as a potent modulator of proteins involved in angiogenesis (VEGF-A), apoptosis, and cell cycle regulation (Bcl-2 and Notch1)^12^. Overexpression of miR-34b suppresses the growth of nasopharyngeal carcinoma cells and mouse tumor xenografts by inhibiting lactate dehydrogenase A, a key enzyme of glycolysis metabolism^38^. MiR-34b/c expression is also correlated with smoking and tumor localization in HNSCC^25^. Recent findings indicate that the antiproliferative, antitumor, anti-inflammatory, antioxidant, and antiangiogenic effects of bioactive compound melatonin in oral cancer cell lines could be exerted through the modulation of miR-34b expression^47^.

Recent advances were made in drug targeting of non-coding RNAs in various diseases. Despite numerous studies demonstrating a key role of microRNAs in cancer, therapeutic risks remain high due to the incomplete complementation of microRNAs with the target sequence that leads to low specificity and unknown/unpredictable adverse effects of miRNA-drugs^48^. MiR-34a was the first microRNA targeted in a clinical trial for cancer treatment (NCT01829971), but the trial was terminated due to serious immune-related adverse events^49^. Nevertheless, the prospects for the future successes of miRNA therapeutics^50^ might be based on the specific delivery of nanotechnology-based drugs at the site of disease, and potentially to personalized approach based on the tissue-specific microRNAs’ DNA methylation and expression or patients' genetic profile.

There are several limitations in our study that could be addressed in future research. The first limitation is the relatively small number of participants, although we broadly confirmed our experimental results on the publicly available data that we used as the validation cohort. However, HPV-negative OSCCs are rare and we did not include other HNSCCs since it is considered a highly heterogeneous disease. Still, the small sample of patients did not negate the potential impact of miR-34b/c as a prognostic biomarker in OSCC. Another possibile limitation is the qualitative assessment of miR-34b/c methylation status. According to the results from the validation TCGA cohort, methylation levels between different CpG sites located in the promotor region highly correlates with each other. The results indicate a clear difference between hypo and hypermethylation of the whole region. All CpG sites have similar contributions in the final methylation status. Finally, we showed a significant difference in miR-34b expression levels between hyper and hypomethylated samples, in both of our cohorts. All these facts indicate that the qualitative method of methylation status assessment used in the study might be a reliable approach for this type of study In clinical setting such as ours, we reasoned that our qualitative but highly sensitive methodology could still be a feasible and practical method for routine diagnostic or prognostic purposes. Also, we selected primer sets that several previous studies established to have high correlation of MS-PCR and Bisulfite Sequencing data^20,37^. Furthermore, we added TCGA analysis of available DNA methylation data as well as microRNA expression data on HPV-negative oral cancer patients. Our TCGA analysis showed high correlation of individual CpG methylation status covered by our MS-PCR primers sets within the miR-34b/c CpG island, and that those 6 CpG are key individual CpGs within the island, with highest clinical and prognostic value. The methylation level of every single analyzed CpG site in the TCGA cohort was in negative correlation with the expression levels of miR-34b. Moreover, integrated multi-dimensional TCGA analysis uncovered miR-34b/c methylation and expression as prognostically informative biomarkers to increase present knowledge on HPV-negative oral cancer. A major limitation might also be the lack of quantitative evaluation of HPV load. However, future studies should address the quantitative methylation analysis and HPV load to obtain a more reliable clinical data.

Only a few studies have conducted the integrated analysis of promoter hypermethylation, gene polymorphisms, and expression of microRNAs. Previous studies, followed by our latest findings, establish mir-34b/c promoter hypermethylation as a potentially valuable prognostic marker in oral carcinomas. As the results from the TCGA cohort have shown, significant differences in expression levels of miR-34b and in the promotor methylation status, between normal and tumoral tissue from the same patients, indicate that those biomarkers might have an important role in the tumorigenesis and tumor progression. Therefore, those molecules and the promotor region might be a potential target for future therapeutics. Especially considering the validated result which demonstrated the significant role of those biomarkers in the overall survival rates of the patients. Since miR-34b/c methylation and miR-34b expression has not been previously investigated in the HPV-negative oral carcinomas, we highlight that the findings of our study might offer new, potentially relevant information for patient management. The findings of our research suggest that miR-34b/c could potentially serve as a therapeutic target in oral cancers. Future studies that involve larger numbers of patients are warranted to evaluate the mir-34b/c DNA methylation and expression as an additional prognostic marker in advanced OSCC and/or HPV-negative oral carcinomas.

## Methods

### MMA hospital cohort subjects

The current study was conducted at the Military Medical Academy (MMA), Belgrade, Serbia, and approved by the Ethics Committee of Military Medical Academy, Belgrade, Serbia (approval number 18/2021). Informed consent was obtained from all individual participants included in the study. All procedures performed in studies involving human tissue samples were in accordance with the ethical standards of the institutional and/or national research committee and with the 1964 Helsinki declaration and its later amendments or comparable ethical standards. All subjects were Caucasians of the same ethnicity. The oral cancer cases were staged according to the TNM classification system for oral and oropharyngeal cancers. General demographic data on MMA Hospital Cohort are given in Table 1. The patients’ median age was 58 (range 39–84), and the median follow-up was 44.4 months (range 8–100 months). None of the patients in our cohort receive radiotherapy or chemotherapy prior to surgery. Tumor tissue was collected immediately after surgical excision, and all patients received radiotherapy, while 44 patients (29.7%) also received postsurgical cisplatin/5-fluorouracil chemotherapy.

### MMA hospital cohort HPV status

Tumor HPV status was determined by the HPV High Risk Screen Real-TM Quant kit (Sacace), and beta-globine gene as an internal control. All OSCC samples were negative for 12 types of HPV (16, 18, 31, 33, 35, 39, 45, 51, 52, 56, 58, 59), including High-risk HPV types (16, 18, 31 and 45), and Intermediate-risk HPV types (33, 35, 39, 51, 52, 56, 58, and 59).

### MMA hospital cohort miR-34b/c promoter hypermethylation status

The DNAs were extracted from tissues of 148 oral carcinomas using TRIZOL reagent (Invitrogen, Germany), and subjected to bisulfite modification with Epitec Bisulfite Kit (Qiagen, Germany). DNA methylation was determined by methylation-specific PCR, with primers specific to discriminate methylated or unmethylated alleles^20,37^. DNA isolated from lymphocytes of healthy individuals were used as unmethylated control and the same DNA treated in vitro with SssI methylase (New England Biolabs, Ipswich, MA, USA) was used as the methylated control.

### MMA hospital cohort miR-34b/c gene expression analysis

microRNAs, isolated from 123 cancer tissue samples by mirVana miRNA Isolation Kit, were reverse-transcribed using the TaqMan microRNA RT kit (Applied Biosystems, USA). The miR-34b/c expression levels were measured by RT-qPCR using TaqMan miRNA assays (Applied Biosystems, USA).

To determine the most suitable endogenous reference gene, in the pilot study of this research we examined gene expression of U6, U44, and U48 in a total of 40 tumor samples. The stability of the candidate reference genes were evaluated with delta Ct method, BestKeeper software^51^, that evaluates the stability reference gene expression, and NormFinder software^52^, which evaluate of the intergroup and intragroup variations. In our analysis, U6 and U44 displayed stability values in close proximity throughout all calculations. While Bestkeaper ranked U44 stability value of 0.612, p = 0.001, as the highest and U6 as the second best with the stability value 0.609, p = 0.001, U6 was ranked the highest by Normfinder, with the lowest inter group variations. Hence, normalization was done using U6 for internal control normalization.The fold changes of miR-34b/c expression were calculated using the 2^−∆∆Ct^ method and normalized to U6. A pool of 5 independent normal oral mucosa specimens from healthy individuals, was used as a calibrator for normalization^50^.

### MMA hospital cohort SNP analysis

Genotyping of rs4938723 T > C polymorphism within the pre-miR-34b/c promoter was conducted in DNA isolated from tumor tissue of 148 OSCC patients and peripheral blood from 175 controls, all Caucasians of the same ethnicity, matched by age and sex. The analysis was carried out by TaqMan SNPs Genotyping Assay (Applied Biosystems, USA).

### TCGA cohort sample selection and data downloading

As a validation cohort in this study, data from The Cancer Genomic Atlas (TCGA) database, Head and Neck Squamous Cell Carcinoma (HNCS) project was used. The query was restricted on white, non-hispanic or latino patients. Only HPV negative, oral cancer cases were analyzed. HPV status of the samples was retrieved from GDAC database, Broad institute (https://gdac.broadinstitute.org/). After the filtration, 225 cases have left and downloaded directly from the server, for further analysis.

### TCGA cohort expression data for miR-34b

The miRNA expression datasets were downloaded for the selected samples. Information about expression of miR-34b/c was extracted for tumor and normal tissue. Data were available for 223 tumor samples and 24 normal tissue samples.

### TCGA cohort methylation data for miR-34b/c promotor region

Methylation array files was retrieved from the TCGA server for selected samples, for tumor and normal tissue. Data was available for 225 tumor samples and 22 normal tissue samples. Methylation array files was downloaded for all selected samples. The region of interest was Chr11:111.512.757-948. In the selected region, 14 CpG sites located in the promotor region of the miR-34b/c genes were examined. Six of them are located in the region experimentally tested in this study. Two CpG sites were excluded from the analysis due to missing values. List of analyzed CpG sites are presented in the Supplementary Fig. 3. In order to determent methylation status of the region (hyper or hypo methylated) for TCGA cohort, K Means Clustering have been performed, using beta values for selected CpG sites for tumor and normal tissue samples.

### Statistical analysis

Data were analyzed by SPSS v.20.0 software (IBM, USA) and JMP Genomics 9.0 statistical software (SAS Institute, USA). The associations between the variables were assessed by the χ^2^ test or Fisher's exact test. A correlation analysis was obtained by Spearman’s correlation test. ROC (Receiver Operating Characteristic) and AUC (Area Under the ROC Curve) analysis was used to determine the optimal cutoff for miR-34b/c expression. The Overall Survival (OS) was determined using the Kaplan–Meier method and the log-rank test, and defined as the time from the surgery until death from any cause or last follow-up. Univariate and multivariate analyses of Cox regression were used to evaluate hazard ratios (HR), with a 95% confidence interval (95% CI). Odds ratios (OR) with 95% CI were estimated by binary logistic regression analysis, with adjustments for gender and age. All reported p values were two-sided, and considered significant when p values were less than 0.05.

## Supplementary Information

Supplementary Information.
