# Supplementary material for: Prognostic impact of miR-34b/c DNA methylation, gene expression, and promoter polymorphism in HPV-negative oral squamous cell carcinomas
Source: Sci Rep. 2022 Jan 25;12:1296. doi: 10.1038/s41598-022-05399-1 (PMC8789922; doi:10.1038/s41598-022-05399-1)
Supplement: Supplementary file 1 — Supplementary Information. [file 41598_2022_5399_MOESM1_ESM.pdf]

Prognostic Impact of miR-34b/c DNA Methylation, Gene Expression, and Promoter Polymorphism in HPV-Negative Oral Squamous Cell Carcinomas

Gordana Supic, Debora Stefik, Nemanja Ivkovic, Ahmad Sami, Katarina Zeljic, Sasa Jovic, Ruzica Kozomara, Danilo Vojvodic, Srboljub Stosic

Supplementary Table S1. Pre-miR-34b/c gene polymorphism association with oral cancer risk.

**pre-miR-34b/c rs4938723**		**OSCC Cases, N=148**	**%**	**Controls, N=175**	**%**	**Adjusted OR [95% CI][a]**	***p***
**Genotype**	**TT**	63	42.57	75	42.86	1	*Ref.* [b]
	**TC**	67	45.27	88	50.29	0.915 [0.573-1.459]	*0.708*
	**CC**	18	12.16	12	6.86	1.780 [0.788-4.019]	*0.165*
**Dominant model**	**TT+TC**	130	87.84	163	93.14	1	*Ref.* [b]
	**CC**	18	12.16	12	6.86	1.865 [0.858-4.055]	*0.116*

Odds Ratio (OR) values are adjusted by age and gender;
Ref. indicates Referent genotype.

Supplementary Figure 1. Receiver operating characteristic (ROC) curve analysis in OSCC cohort. Area under the ROC curve (AUC) values of 0.654, [0.552-0.756], p=0.004, indicated that fold change expression of 4.54 is an optimal cutoff point with highest prognostic performance in our cohort.

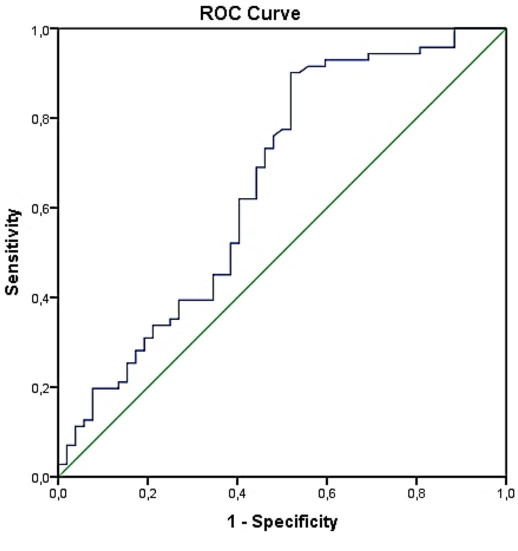

Supplementary Figure 2.

K Mean Clustering – methylation status determination

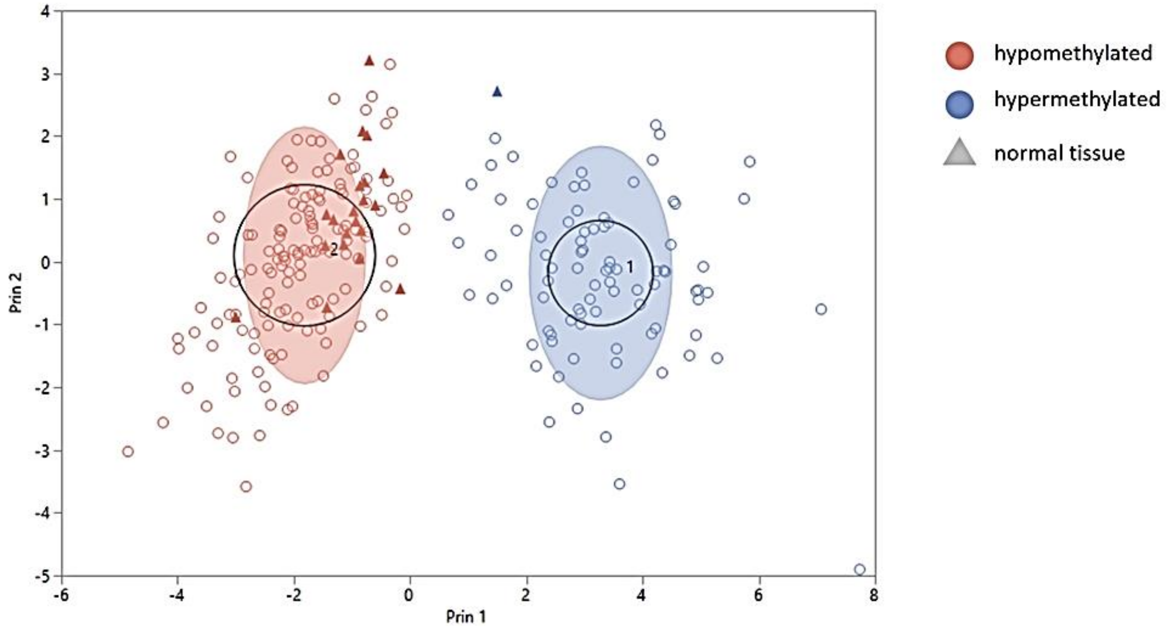

Supplementary Figure 3.

p<0.0001 For each showed correlation

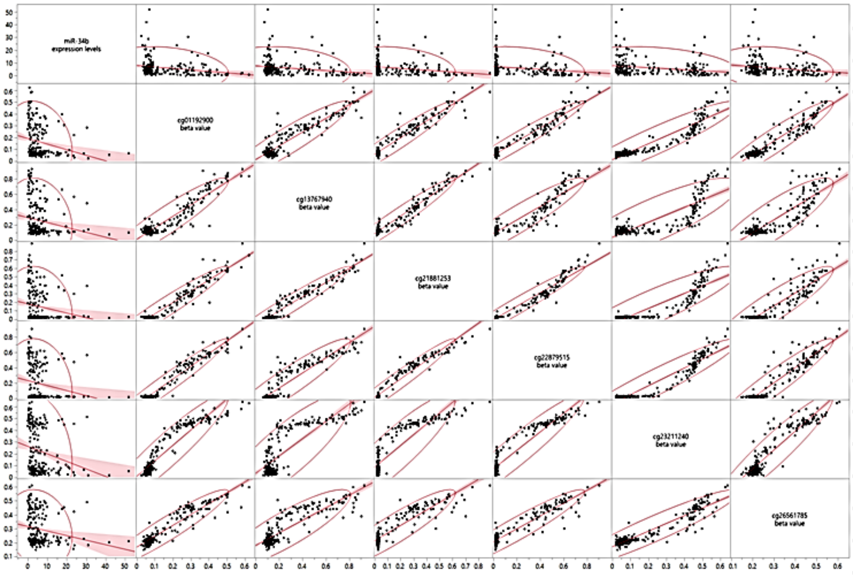

Variable	by Variable	Spearman ρ	Prob>|ρ|
cg23211240 beta value	miR-34b expression levels	-0.3324	<.0001
cg01192900 beta value	miR-34b expression levels	-0.3243	<.0001
cg22879515 beta value	miR-34b expression levels	-0.2905	<.0001
cg13767940 beta value	miR-34b expression levels	-0.2899	<.0001
cg26561785 beta value	miR-34b expression levels	-0.2829	<.0001
cg21881253 beta value	miR-34b expression levels	-0.2607	<.0001
cg26561785 beta value	cg23211240 beta value	0.8639	<.0001
cg26561785 beta value	cg22879515 beta value	0.7957	<.0001
cg23211240 beta value	cg22879515 beta value	0.8645	<.0001
cg26561785 beta value	cg21881253 beta value	0.7956	<.0001
cg23211240 beta value	cg21881253 beta value	0.7998	<.0001
cg22879515 beta value	cg21881253 beta value	0.8787	<.0001
cg23211240 beta value	cg13767940 beta value	0.7192	<.0001
cg26561785 beta value	cg13767940 beta value	0.7447	<.0001
cg22879515 beta value	cg13767940 beta value	0.8182	<.0001
cg21881253 beta value	cg13767940 beta value	0.8407	<.0001
cg13767940 beta value	cg01192900 beta value	0.6731	<.0001
cg21881253 beta value	cg01192900 beta value	0.7487	<.0001
cg22879515 beta value	cg01192900 beta value	0.817	<.0001
cg26561785 beta value	cg01192900 beta value	0.846	<.0001
cg23211240 beta value	cg01192900 beta value	0.9191	<.0001
